# The developmental lipidome of *Nippostrongylus brasiliensis*

**DOI:** 10.1186/s13071-024-06654-2

**Published:** 2025-01-25

**Authors:** Tao Wang, Michael G. Leeming, Nicholas A. Williamson, Tiffany Bouchery, Rory Doolan, Graham Le Gros, Gavin E. Reid, Nicola L. Harris, Robin B. Gasser

**Affiliations:** 1 Department of Veterinary Biosciences, Melbourne Veterinary School, Faculty of Science, The University of Melbourne, Parkville, VIC 3010 Australia; 2 Bio21 Mass Spectrometry and Proteomics Facility, The University of Melbourne, Parkville, VIC 3010 Australia; 3 Department of Medical Parasitology and Infection Biology, Swiss Tropical and Public Health Institute, Kreuzstrasse 2, CH-4123 Allschwil, Switzerland; 4 Malaghan Institute of Medical Research, Kelburn, Wellington, 6012 New Zealand; 5 School of Chemistry, The University of Melbourne, Parkville, VIC 3010 Australia; 6 Department of Biochemistry and Pharmacology, The University of Melbourne, Parkville, VIC 3010 Australia; 7 Bio21 Molecular Science and Biotechnology Institute, The University of Melbourne, Parkville, VIC 3010 Australia; 8 Department of Immunology, School of Translational Medicine, Monash University, Melbourne, VIC Australia

**Keywords:** *Nippostrongylus brasiliensis*, Gastrointestinal nematode, Rodent, Lipidome, Lipids, Mass spectrometry, Adaptation

## Abstract

**Background:**

*Nippostrongylus brasiliensis*—a nematode of rodents—is commonly used as a model to study the immunobiology of parasitic nematodes. It is a member of the Strongylida—a large order of socioeconomically important parasitic nematodes of animals. Lipids are known to play essential roles in nematode biology, influencing cellular membranes, energy storage and/or signalling.

**Methods:**

The present investigation provides a comprehensive, untargeted lipidomic analysis of four developmental stages/sexes (i.e. egg, L3, adult female and adult male stages) of *N. brasiliensis* utilising liquid chromatography coupled to mass spectrometry.

**Results:**

We identified 464 lipid species representing 18 lipid classes and revealed distinct stage-specific changes in lipid composition throughout nematode development. Triacylglycerols (TGs) dominated the lipid profile in the egg stage, suggesting a key role for them in energy storage at this early developmental stage. As *N. brasiliensis* develops, there was a conspicuous transition toward membrane-associated lipids, including glycerophospholipids (e.g. PE and PC) and ether-linked lipids, particularly in adult stages, indicating a shift toward host adaptation and membrane stabilisation.

**Conclusions:**

We provide a comprehensive insight into the lipid composition and abundance of key free-living and parasitic stages of *N. brasiliensis*. This study provides lipidomic resources to underpin the detailed exploration of lipid biology in this model parasitic nematode.

**Graphical Abstract:**

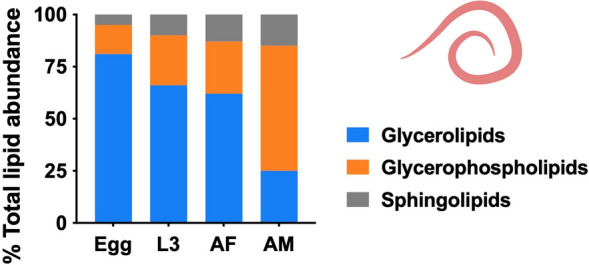

**Supplementary Information:**

The online version contains supplementary material available at 10.1186/s13071-024-06654-2.

## Background

Gastrointestinal parasitic nematodes are responsible for numerous human and animal diseases, exerting significant impacts on global health and agriculture [1, 2]. For instance, the soil-transmitted nematodes *Ancylostoma duodenale* and *Necator americanus* (hookworms) infect ~ 472 million people globally, contributing to an estimated disease burden of 4 million disability-adjusted life years (DALYs) [3]. In the livestock industry, *Haemonchus contortus* and related strongylid nematodes are widespread and affect animals globally [2]. Currently, the primary method for controlling parasitic nematode infections relies on chemical treatment. However, the frequent use of anthelmintic drugs has led to widespread resistance in an increasing number of nematode species [4–6]. This issue has driven the search for alternative control strategies, such as novel drugs with different modes of action or vaccines [7–9].

Lipids play critical roles in the biology of parasitic nematodes, particularly in relation to cellular membrane structure, energy storage and both intra- and inter-cellular signalling. Previous studies of lipids in parasitic nematodes were constrained by using low-throughput methods, such as thin-layer and/or gas chromatography, which identified only small numbers of fatty acids (*n* = 9–31) in a small number of nematode species, including *Ascaris lumbricoides* [10], *Brugia malayi* [11], *Dictyocaulus viviparus* [12] and *Strongyloides ratti* [13]. Fortunately, mass spectrometry-based lipidomics now offers a powerful analytical platform, enabling the comprehensive characterisation of lipid composition and abundance [14, 15]. This approach not only enhances our understanding of the lipids themselves but also will likely provide valuable biochemical and molecular insights into host-parasite interactions and disease processes. To date, targeted or untargeted lipidomic studies have been conducted on parasitic nematode species of significant socioeconomic importance, such as *Ascaris suum*, *H. contortus*, *Onchocerca volvulus* and *Toxocara canis* [16–19] as well as *Heligmosomoides polygyrus* of rodents [20].

*Nippostrongylus brasiliensis*—also a gastrointestinal nematode of rodents—serves as a useful model for studying host-parasite interactions and the immunobiology of nematodes [21–23]. While extensive research has been conducted on host immune responses to *N. brasiliensis* [24, 25], limited information is available on its lipid composition in different developmental stages of this nematode. In this study, we characterised the lipidome of four developmental stages/sexes of *N. brasiliensis* using liquid chromatography tandem mass spectrometry (LC-MS/MS). Defining the lipidome of *N. brasiliensis* will assist in elucidating the lipid biology of this parasite, both in the host animal and in the external environment.

## Methods

### Parasite material

In total, four key developmental stages [i.e. egg; third-stage (L3) larvae; female (AF) and male adults (AM)] of *N. brasiliensis* were produced in Sprague-Dawley rats (purchased from Animal Resources Centre, Perth, Australia) as described previously [21]. Briefly, rats were injected subcutaneously with 3000 infective L3s of *N. brasiliensis*. Eggs were collected from the faeces from infected animals (7 days after injection). Adult worms were recovered using the Baermann technique [26] from the lung tissues and small intestines of rats 8 days after inoculation. Male and female adults of *N. brasiliensis* were separated. For each stage/sex, four biological replicates of each life stage were collected, washed extensively (five times) in large volumes of physiological saline (pH 7.0), pelleted and then frozen at – 80 °C until further analysis. All animal experiments were approved by the animal ethics committee of Monash University, Campus, Melbourne, Australia (authorization number: E/1843/2018/M).

### Tissue homogenisation and lipid extraction

Four replicate samples for each stage or sex (egg, L3, AF and AM) were lyophilised in a benchtop manifold freeze-drier prior to homogenisation and extraction. Freeze-dried samples (~ 4 mg each) were individually transferred to an Eppendorf tube (1.5 ml) containing 200 μl ice-cold 40% (*v/v*) methanol, with 10 μl isotope-labelled internal lipid standards solution (330710X, Mouse SPLASH^®^ LIPIDOMIX, Merck, USA). Samples were homogenised in 100 μl containing 0.5-mm zirconium oxide beads (ZROB05, Next Advance, USA) in a blender (Bead Bullet GOLD, Next Advance, USA) at speed 10 for 30 s, eight cycles. Blank tubes containing 100 µl water (included as controls) were processed in the same manner.

Then, lipids were extracted using an established monophasic lipid extraction protocol [27]. In brief, 800 μl water/chloroform/methanol (0.74/2/1, *v/v/v*) was added to each tube. The tubes were vortexed for 60 s and incubated on a ThermoMix (Eppendorf, Germany) at 1000 × *g* for 30 min at 22 °C and then centrifuged at 14,000 × *g* for 15 min at 4 °C. The supernatant was transferred to another tube, and 400 μl chloroform/methanol (1/2, *v/v*) was added, vortexed (10 s) and centrifuged, as before. The supernatant was collected and combined with the supernatant from the first extraction. Individual lipid samples were then dried in a SpeedyVac, and each lipid pellet was re-suspended in 100 μl methanol/chloroform (9/1, v/v) prior to analysis.

### High-performance liquid chromatography (HPLC) and mass spectrometric (MS) analyses

All four samples (four replicates for each) were analysed by ultrahigh performance liquid chromatography (UHPLC) employing a Vanquish UHPLC, coupled to an Orbitrap Fusion Lumos mass spectrometer (Thermo Fisher Scientific, USA), with separate runs in positive and negative ionisation mode polarities. Solvent A was 6/4 (v/v) acetonitrile/water containing 5 μM medronic acid, and solvent B was 9/1 (v/v) isopropanol/acetonitrile; both solvents A and B contained 10 mM ammonium acetate. Each sample (10 μl) was injected into an Acquity UPLC HSS T3 C18 column (1 × 150 mm, 1.8 µm; Waters, USA) at 50 °C at a flow rate of 60 μl/min for 3 min using 3% solvent B. Gradient elution was conducted over 33 min with the following solvent timetable: (time in min, %B): (0, 3), (5, 3), (10, 70), (26, 99), (29, 99), (29.1, 3), (33, 3).

All MS experiments were performed using a heated electrospray ionisation (HESI) source. The spray voltages were 3.5 kV in positive ionisation mode and 3.0 kV in negative ionisation mode. In both polarities, the flow rates of sheath, auxiliary and sweep gases were 25 and 5 and 0 ‘arbitrary’ unit(s), respectively. The ion transfer tube and vapouriser temperatures were maintained at 300 °C and 150 °C, respectively, and the S-Lens RF level was set at 50%. In the positive ionisation mode from 5 to 33 min, a top speed data-dependent scan with a cycle time of 1 s was used. Within each cycle, full-scan MS-spectra were acquired first in the Orbitrap at a mass resolving power of 120,000 (at m/z 200) across an m/z range of 300–2000 using quadrupole isolation, an automatic gain control (AGC) target of 4e5 and a maximum injection time of 50 ms, followed by HCD-MS/MS at a mass resolving power of 15,000, a normalised collision energy (NCE) of 27% in positive ionisation mode and 30% in negative ionisation mode, an m/z isolation window of 1, a maximum injection time of 35 ms and an AGC target of 5e4. To characterise glycerophosphocholine (PC) lipid cations, a data-dependent product ion (m/z 184.0733)-triggered collision-induced dissociation (CID)-MS/MS scan was performed in the cycle using a q-value of 0.25 and a NCE of 30%, with other settings being the same as that for HCD-MS/MS. For the characterisation of TG lipid cations, the (fatty acid + NH_3_) neutral loss product ions observed by HCD-MS/MS were used to trigger the acquisition of top-3 data-dependent CID-MS3 scans in the cycle using a q-value of 0.25 and a NCE of 30%, with other settings being the same as that for HCD-MS/MS.

### Identification and quantification of lipids and statistical analysis

LC-MS/MS data were searched for lipids via MS-DIAL 4.80 [28]. The mass accuracy settings were 0.005 Da and 0.025 Da for MS1 and MS2. The minimum peak height was 50,000 and mass slice width was 0.05 Da. The identification score cutoff was 80%. Post-identification processing was done with a text file containing the name and m/z of each standard in the SPLASH^®^ LIPIDOMIX^®^ Mass Spec Standard (330710X, Avanti Polar Lipids, USA). In positive ionisation mode, [M + H]^+^, [M + NH_4_]^+^ and [M + H-H2O]^+^ were selected as ion forms. In negative ionisation mode, [M-H]^−^ and [M + CH_3_COO]^−^ were selected as ion forms. All lipid classes available were selected for the search. The retention time tolerance for alignment was 0.1 min. Lipids with maximum intensity less than fivefold the average intensity in the blank sample were removed. All other settings were set as the defaults. All lipid LC-MS features were manually inspected and re-integrated when needed. Four types of lipids, (i) lipids with only sum composition except SM, (ii) lipid identifications due to peak tailing, (iii) retention time outliers within each lipid class and (iv) LPA and PA artefacts generated by in-source fragmentation of LPS and PS, were also removed. The shorthand notation used for lipid classification and structural representation follows the nomenclature proposed previously [29].

Relative quantification of lipid species was achieved by normalisation of the LC peak heights of identified lipids against the peak heights and concentrations of the corresponding internal lipid standards from the same lipid class.

Semi-quantitative analysis of lipid species was achieved by comparing the identified peak areas against those of the correspondent isotope labelled internal lipid standards in the same lipid class, including diradylglycerols (DG), triradylglycerols (TG), glycerophosphatidic acid (PA), glycerophosphocholines (PC), glycerophosphoethanolamines (PE), glycerophosphoglycerols (PG), glycerophosphoinositols (PI), glycerophosphoserines (PS), lysoPC (LPC), lysoPE (LPE) and SM lipids, and reported as the amount of lipid per mg of dry weight. For lipid classes or sub-classes without correspondent stable isotope-labelled lipid internal standards, the LC peak areas of individual molecular species within these classes were normalised as follows: monoradylglycerol (MG) species against DG (18:1D7_15:0); cardiolipin (CL) and lysoPG (LPG) against PG (18:1D7_15:0); lysoPA (LPA) against PA(18:1D7_15:0), lysoPS (LPS) against PS (18:1D7_15:0) and ceramide (Cer) against SM (d36:2D9). Given that the commercial availability of some stable isotope-labelled lipid standards is limited, some of the identified lipids were normalised against a standard from a different class or sub-class. No attempts were made to quantitatively correct for different ESI responses of individual lipids due to concentration, acyl chain length, degree of unsaturation or matrix effects caused by differences in chromatographic retention times compared with the relevant standards. The results reported here are, therefore, for relative quantification and should not be considered to reflect the absolute concentrations of each lipid or lipid sub-class [30].

### Bioinformatic analyses

Principal component analysis (PCA) was conducted using Perseus software (v.1.6.1.1) [31]. An UpSet plot of lipidomes was produced using Intervene [32]. One-way ANOVA Post Hoc Test was performed for multiple group comparisons by using GraphPad Prism 10.2.3 software (GraphPad, La Jolla, USA). Error bars represent standard deviation (SD); statistical significance was set at *P* < 0.05.

## Results

### Lipidome of developmental stages

In total, 464 lipid species representing 18 lipid classes or sub-classes within three lipid categories—including glycerolipids (GL), glycerophospholipids (GP) and sphingolipids (SP)—were confidently identified and quantified in the four key developmental stages/sexes of *N. brasiliensis* (Table 1 and Table S1). For all samples, lipid species from the GL and GP categories were most abundant, accounting for 46% (*n* = 214) and 49% (*n* = 228) of all lipid species identified, respectively. TG (*n* = 179) dominated the GL category, whereas in the GP category, the most commonly identified classes were PE (*n* = 67), PI (*n* = 41) and PC (*n* = 22).

**Table 1:** Summary of the numbers of lipid species identified in lipid extracts from different developmental stages/sexes of *Nippostrongylus brasiliensis*

Lipid category/class*	Egg	L3	AF	AM	Total identified lipids
Glycerolipids					
MG	3	3	2	3	3
DG	30	25	25	22	32
TG	170	124	166	95	179
Glycerolipids					
PA	4	0	2	2	5
PC	21	17	21	18	22
PE	60	45	54	45	67
PG	6	5	5	4	5
PI	37	12	30	18	41
PS	13	12	10	9	17
CL	15	5	7	6	16
LPA	2	0	1	1	2
LPC	18	11	14	12	18
LPE	13	12	12	12	17
LPG	4	4	3	4	4
LPI	7	2	6	5	7
LPS	7	5	1	1	7
Sphingolipids					
SM	6	6	6	5	6
Cer	16	14	16	16	16
Totals	432	302	381	278	464

^*^MG monoradylglycerols, *DG* diradylglycerols, *TG* triradylglycerols, *PA* glycerophosphatidic acid, *PC* glycerophosphocholines, *PE* glycerophosphoethanolamines, *PG* glycerophosphoglycerols, *PI* glycerophosphoinositols, *PS* glycerophosphoserines, *CL* cardiolipins. For LPA, LPC, LPE, LPG, LPI and LPS, prefix 'L' was added for each lysophospholipid class, *SM* sphingomyelins, *Cer* ceramide

**Fig. 1:**
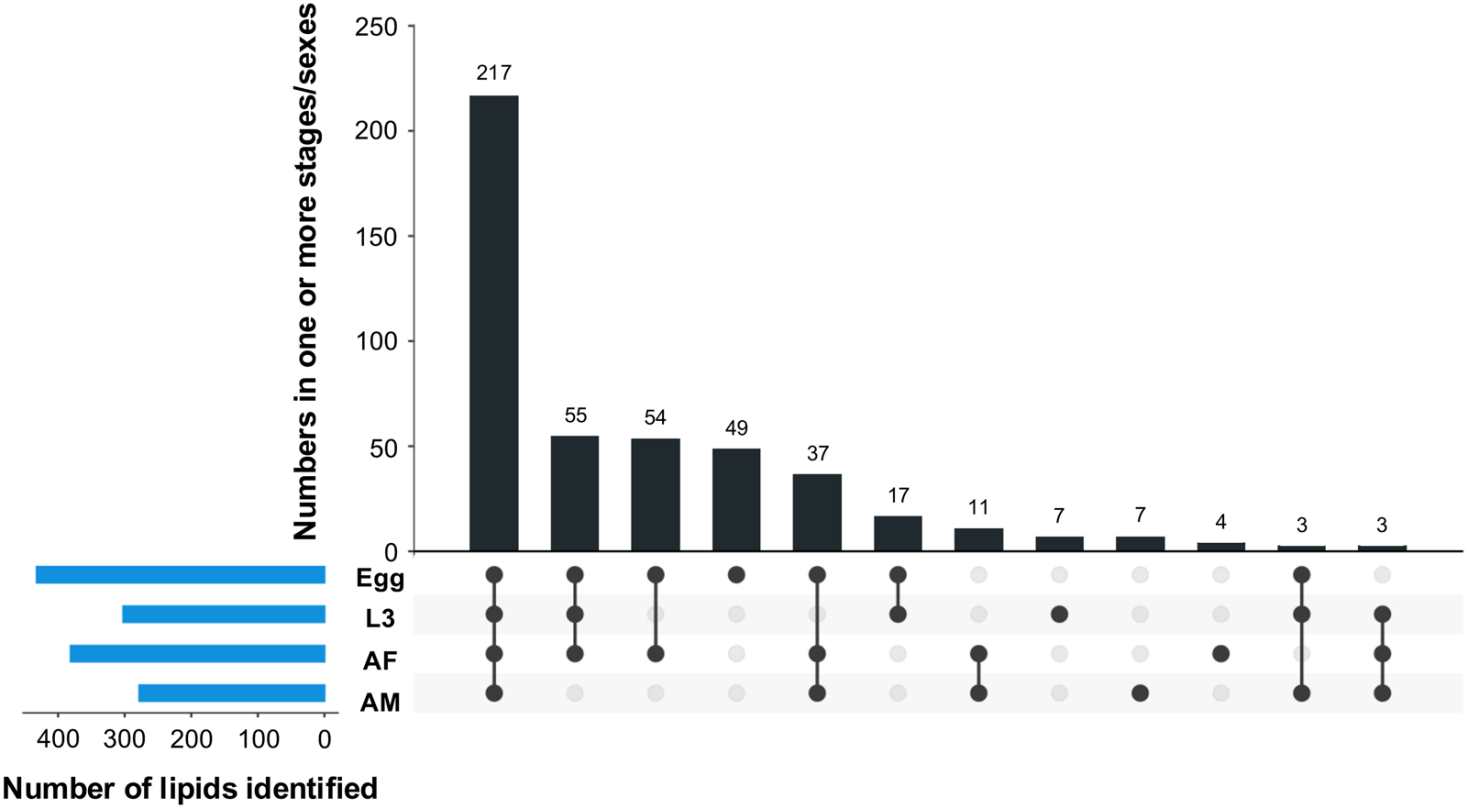
Presence and numbers of identified lipids in the egg, third larval (L3) and female (AF) and male adult (AM) stages. Connected dots show the shared lipid species between or among developmental stages/sexes, and the total number of lipid species in a particular stage/sex is shown in the bar size

Most lipids were identified in the egg stage (*n* = 432), followed by AF (*n* = 381), L3 (*n* = 302) and AM (*n* = 278) (Table 1). Lipid species common to developmental stages/sexes (i.e. egg, L3, AF, AM) are shown in Fig. 1. Most lipid species (*n* = 397, 86%) were present in at least two developmental stages/sexes, with 217 species (47%) identified in all stages/sexes. In contrast, a markedly smaller number of lipid species (*n* = 67, 14%) was uniquely identified in a single stage or sex. The egg stage contained most unique lipid species (*n* = 49, 11%), followed by L3 and AM, both with seven unique lipids (2%), whereas AF had four unique lipids. A comprehensive list of identified lipid species for each life stage/sex is provided in Table S1.

### Fatty acyl composition of the lipidome

An analysis of fatty acyl compositions revealed that the lipidome of *N. brasiliensis* is rich in unsaturated, even-carbon numbered and long-chain fatty acyls (> 12 carbons), representing 58%, 83% and 99% of the total fatty acyl compositions, respectively (Table 2). In total, 74 saturated lipid species in 13 classes were detected, with the highest numbers being TG (*n* = 19), followed by PE (*n* = 15) and LPE (*n* = 8) (Table S1). Additionally, 58 ether-linked lipids were linked to the GP category, including PE (*n* = 33), PI (*n* = 10), PC (*n* = 9), PS (*n* = 4) and LPC (*n* = 2) (Table S1).

**Table 2:** Fatty acyl (FA) composition of lipid species identified in the lipidome of *Nippostrongylus brasiliensis*

Lipid category	Saturated (%)		Unsaturated (%)		Odd-chain FA (%)	Even-chain FA (%)	Total no. of FAs
	Medium-chain FA ^a^	Long-chain FA^b^	Medium-chain FA	Long-chain FA			
Glycerolipids	1.5	40.6	ND^c^	57.9	17.9	82.1	604
Glycerophospholipids	ND	40.9	ND	59.1	13.6	86.4	411
Sphingolipids	ND	63.9	ND	36.1	52.8	47.2	36
Totals	0.9	41.5	ND	57.7	17.4	82.6	1051

^a^Medium-chain FA contains 6–12 carbons

^b^Long-chain FA contains > 12 carbons

^c^ND, not detected

### Alterations in lipid abundance during the development of *N. brasiliensis*

**Fig. 2:**
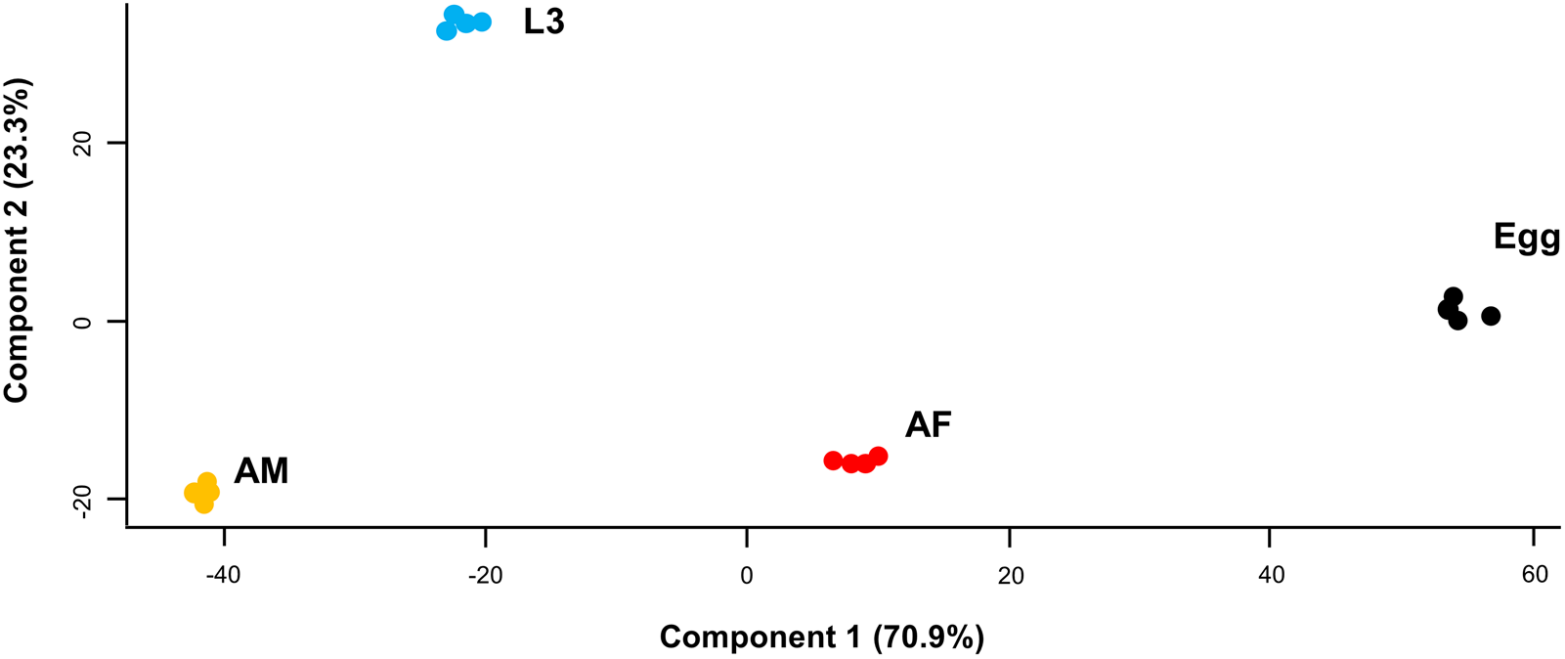
Principal component analysis of the developmental lipidome of *Nippostrongylus brasiliensis* representing egg; third larval (L3); female (AF) and male adult (AM) stages

Principal component analysis (PCA) of the developmental lipidome revealed that differences in lipid quantity among the four developmental stages (i.e. egg, L3, AF and AM) were greater than the variation within a single stage (i.e. among all four replicates) (Fig. 2). The two-dimensional plot clearly separated the lipidomic data into three distinct groups: egg, L3 and the adult stages (i.e. AF and AM).

**Fig. 3:**
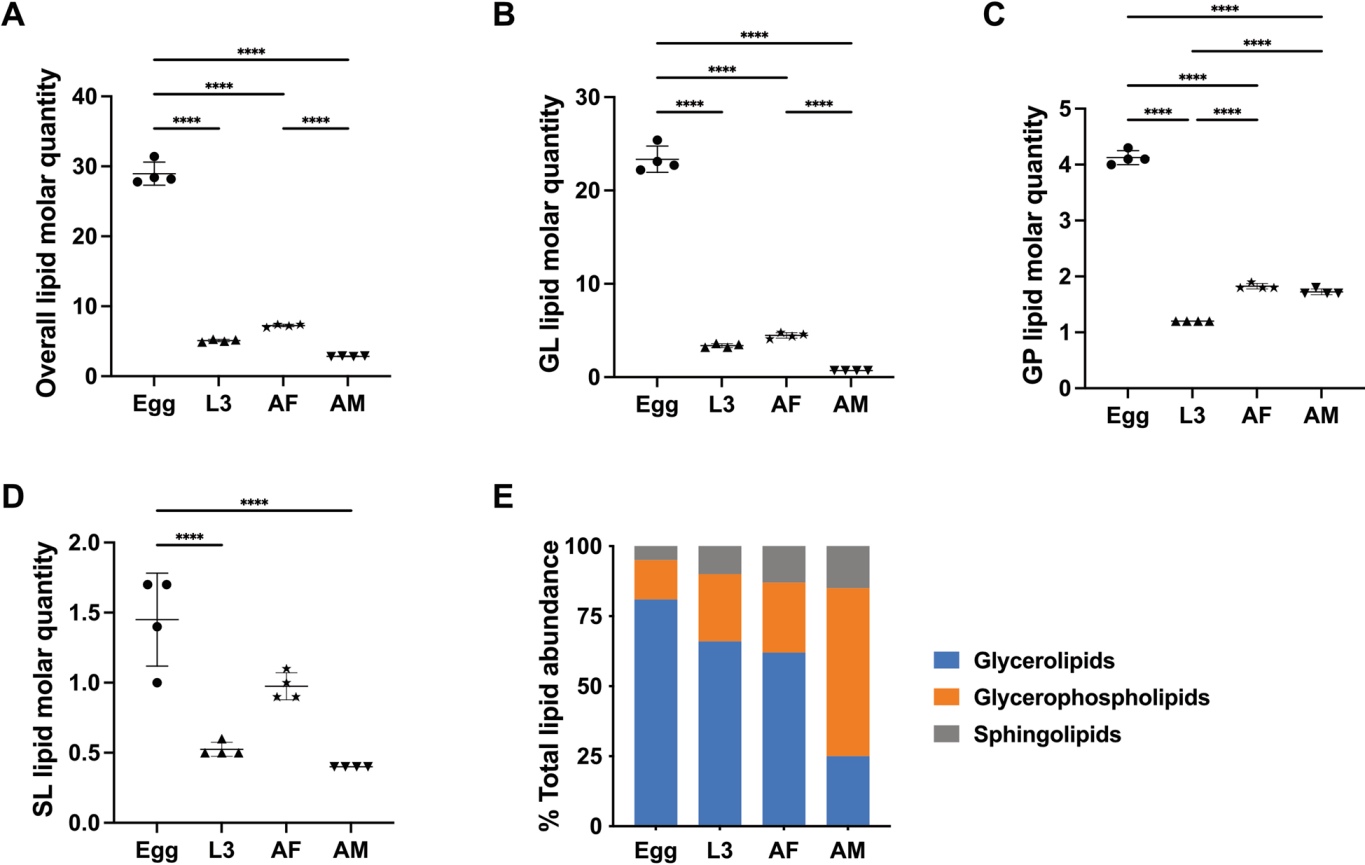
Quantification changes of lipids overall, as well as the glycerolipid (GL), glycerophospholipid (GP) and sphingolipid (SL) categories and the proportions of these categories in different developmental stages/sexes of *Nippostrongylus brasiliensis*. Four developmental stages/sexes include egg, third larval (L3); female (AF) and male (AM) adult stages (x-axis). Statistical analysis was performed by ANOVA (only significant results with ****P < 0.0001; details are provided in Additional file 2: Table S3). Error bars indicate standard deviation (four replicates)

**Fig. 4:**
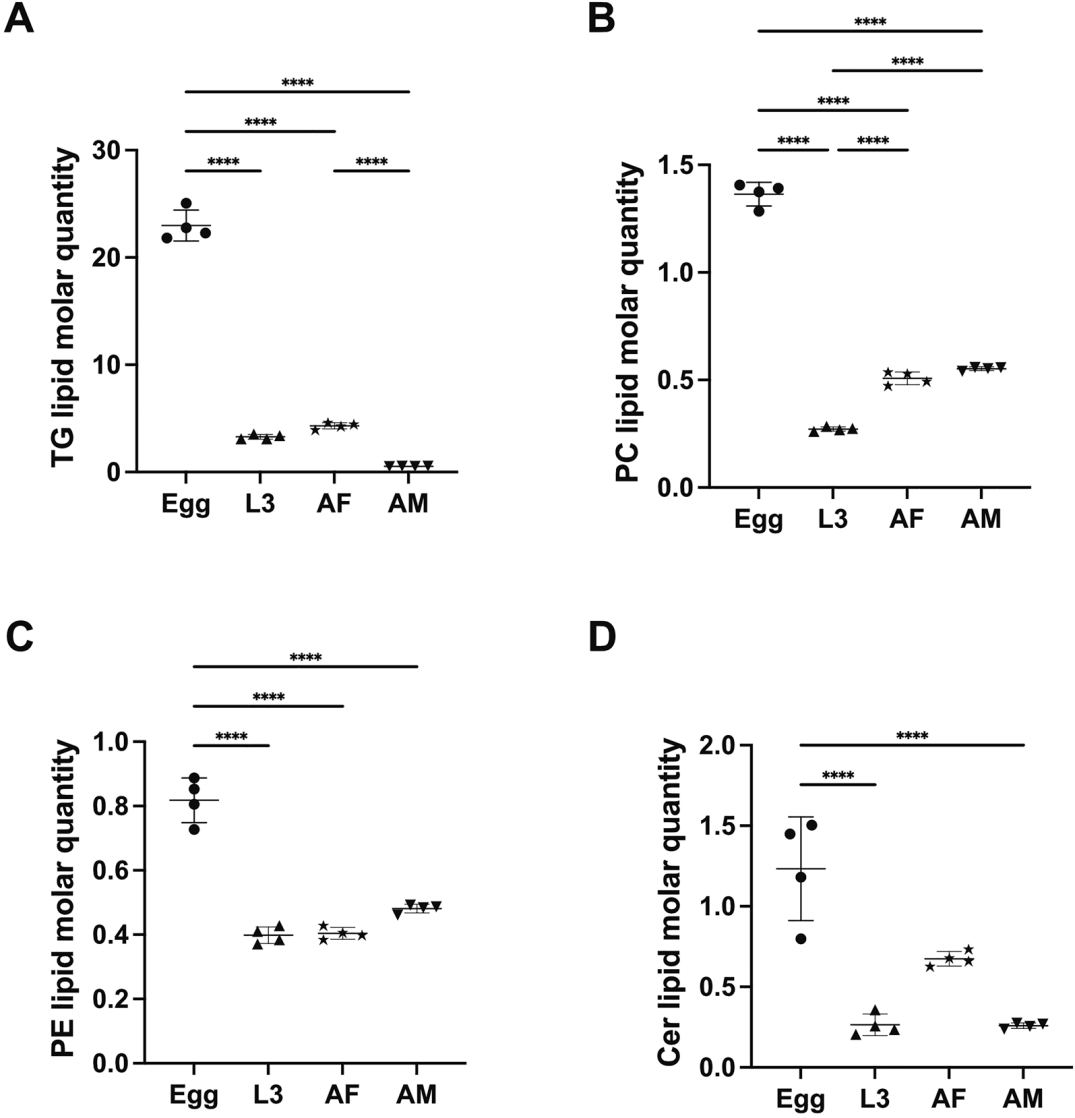
Quantitative changes of saturated and ether-linked lipid species in different developmental stages/sexes of *Nippostrongylus brasiliensis*. Four developmental stages/sexes include egg, third larval (L3); female (AF) and male (AM) adult stages. Statistical analysis was performed by ANOVA (only significant results with ****P < 0.0001; details are provided in Additional file 2: Table S3). Error bars indicate standard deviation (four replicates)

Of all developmental stages/sexes studied, the highest total lipid concentration was observed in the egg stage, with > 28 μmole/mg (micromoles of lipids per milligram of dry body weight), whereas other stages (i.e. L3, AF and AM) contained < 8 μmole/mg (Fig. 3A). The GL category, dominated by numerous TG species, contributed significantly to the lipid abundance in the egg, L3 and AF stages, whereas GP was the most abundant lipid category in the AM stage (Fig. 3). Lipid classes such as TG, PC, PE and Cer were consistently present in high abundance across all four developmental stages/sexes (Fig. 4). At the individual lipid species level, TG species including TG (52:1), TG (52:2), TG (54:2), TG (54:3) and TG (54:6), with even-numbered fatty acyl chains (e.g. 16:0, 18:0, 18:1, 18:2 or 20:4), were dominant across all stages. Many of these lipid species (*n* = 13) had high lipid concentrations (> 0.5 μM/mg) in the egg stage (Table S1).

**Fig. 5:**
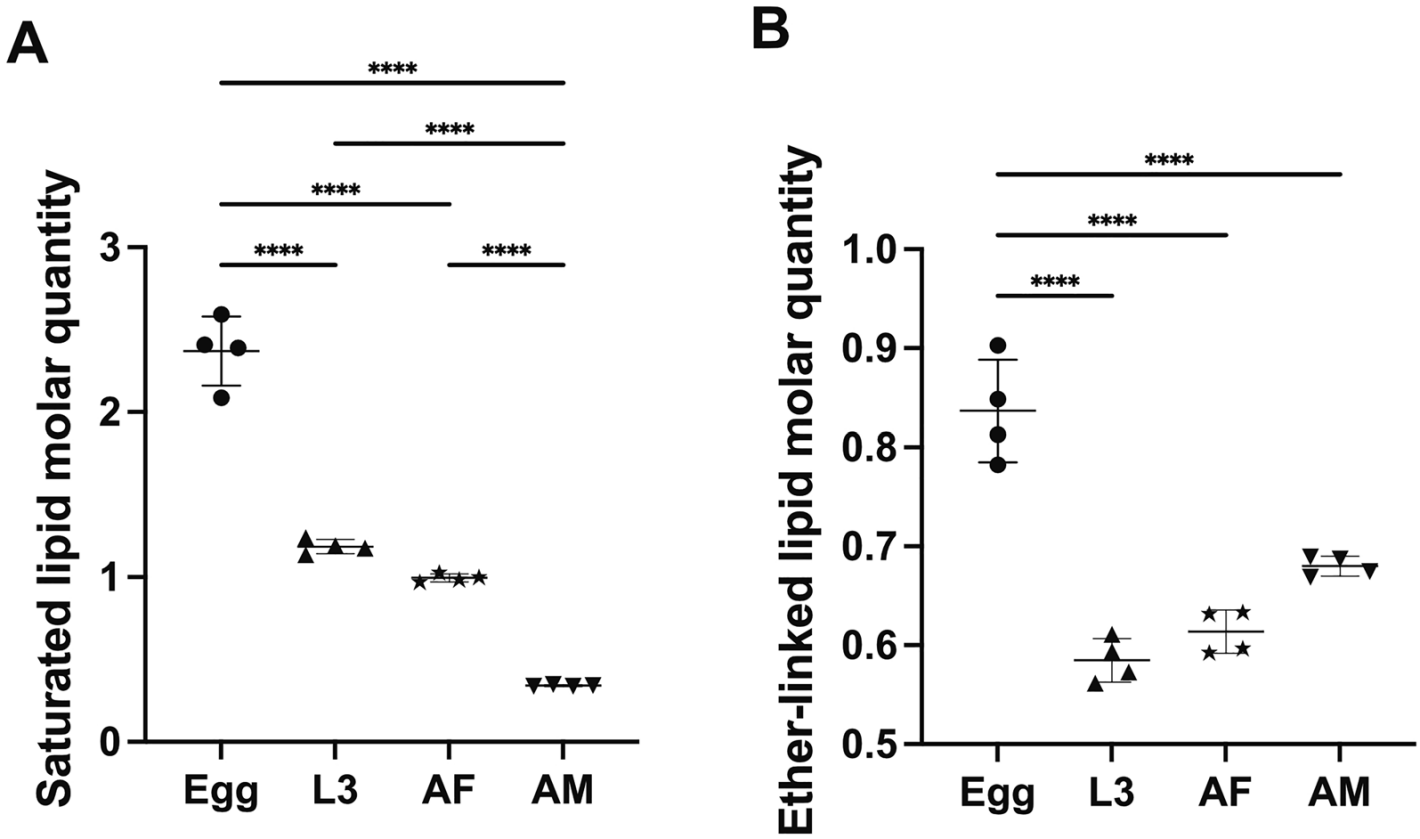
Quantitative changes of TG, PC, PE and Cer lipid classes in different developmental stages/sexes of *Nippostrongylus brasiliensis*. Four developmental stages/sexes include egg, third-stage (L3) larvae; female (AF) and male (AM) adult stages. Statistical analysis was performed by ANOVA (only significant results with ****P < 0.0001; details are provided in Additional file 2: Table S3). Error bars indicate standard deviation (four replicates)

A closer examination of the lipid category abundance (as a percentage of total lipid abundance in a particular developmental stage/sex) revealed a substantial increase of membrane structure- and signaling-related GP and SL lipids during development (Fig. 3E). Specific lipids, such as PG (36:1), LPE (18:0), PE (36:2e), PC (34:3) and PC (36:3), which contain C18 fatty acyl chains (e.g. 18:0, 18:1 and 18:2), were key contributors to the upregulation of overall GP abundance (Table S1). Although saturated and ether-linked lipids were prevalent in the egg stage (Fig. 5), they only represented a small proportion of the total lipidome (8% and 2%, respectively). By comparison, saturated lipids accounted for 12–23% of the total lipidome in the L3, AF and AM stages, whereas ether-linked lipids made up 9–24% of the lipidome in these stages. The full list of lipid class and the alterations of abundance of individual lipid species during development are presented in Fig. S1 and Table S1.

## Discussion

A previous lipidomic study of the infective L3 stage of *N. brasiliensis* provided an important context for understanding the lipid metabolism of this model nematode [33]. Yeshi et al. (2020) revealed 332 key lipid species that are predominantly involved in energy storage and membrane composition during this infective phase. The present findings align with those of this previous study [33]. Using LC-MS/MS, our study provides a comprehensive developmental lipidomic characterisation of *N. brasiliensis* for key developmental stages/sexes: eggs, L3 and adult worms (i.e. AF and AM). The identification of 464 lipid species in 18 lipid classes or sub-classes highlights the complexity and importance of lipid metabolism in this nematode. Notably, energy storage-related GL and membrane stability-related GP were the dominant lipid categories, consistent with published findings for some other nematode species including *H. contortus* and *A. suum*, where similar lipid profiles likely support energy storage and membrane integrity during development [17, 18].

The lipid profile of *N. brasiliensis* reveals distinct shifts during development, which likely reflect the parasite's need to adapt to varying environmental and physiological changes and conditions. A key finding was the dominance of TG in the egg stage, constituting a major portion of the lipidome. This observation accords with studies of other nematode species, in which TG serves as a critical energy reserve during embryogenesis and early larval development [34]. The high TG content in eggs suggests that these lipids are mobilised to fuel cellular processes during early development when external nutrient sources are limited or unavailable. This reliance on TG for early development has been observed in *H. contortus*, where TGs were found to be critical in the free-living larval stages prior to infection of the host animal [17].

As the nematode transitions from the egg to L3 and adult stages, the lipid profile shifts markedly. The reduction in TG in later stages, particularly in adult males, suggests that the parasite’s energy metabolism becomes more dependent on host-derived nutrients rather than internal reserves. This interpretation is further supported by the increased abundance of membrane lipids, such as GP, in the adult stages. These lipids, particularly PE and PC, play critical roles in maintaining membrane structure and cellular signalling in parasite worms such as *H. contortus* and *Schistosoma mansoni* [17, 35]. The shift from energy storage to membrane remodelling and signal transduction likely reflects the parasite’s adaptation to its environment within the host animal. Notably, female adult *N. brasiliensis* contained significantly higher amounts of TG than male adults (Fig. 5), which likely associates with the high TG content in eggs within the reproductive tract of gravid female worms and is consistent with previous findings for *H. contortus* [34].

Another notable difference in the *N. brasiliensis* developmental lipidome is the higher proportion of ether-linked lipids in the L3 (12%), AF (8%) and AM (24%) stages compared with the egg stage (3%), with the highest percentage recorded in AM. Ether-linked lipids, such as ether-linked PE, are well recognised for their role in enhancing membrane stability and in protecting cells from oxidative damage [36, 37]. This feature is particularly critical for the infective L3 stage of *N. brasiliensis* during host invasion, where the larvae face significant environmental stresses, including temperature fluctuations and exposure to the host’s immune system. The increased abundance of these lipids in L3 likely reflects an adaptation to enhance membrane resilience, facilitating the larvae’s survival during the critical transition to parasitism [17]. Furthermore, ether-linked lipids contribute to reducing the permeability of membranes, which could help *N. brasiliensis* L3 withstand oxidative stress and immune attack as it migrates through host tissues. In the adult stages, particularly AM, the high abundance of these lipids might support their survival during nutrient uptake in tissues, when exposure to reactive oxygen species (ROS) from the host immune response is heightened [38]. Similar lipid adaptations have been observed in other parasitic worms, such as *S. mansoni*, where ether-linked lipids in the tegumental membrane provide enhanced protection against host-derived oxidative stress [39]. This information suggests that ether-linked lipids in *N. brasiliensis* play a dual role—they not only maintain membrane integrity but also offer crucial protection against the host’s immune defenses, particularly in the later developmental stages when the parasite resides in a more hostile host environment.

## Conclusions

This study reports the comprehensive developmental lipidomic profile for *N. brasiliensis*, revealing significant lipidomic differences among key developmental stages. Lipids essential for energy storage (TG) dominate the early stages, whereas membrane-related lipids (e.g. PE and PC) and ether-linked lipids become more prominent in adults and likely support host adaptation and survival. Overall, these findings and the lipidomic resources developed here provide a sound basis for understanding lipid biology in this model parasitic nematode.

## Supplementary Information

Additional file 1: Table S1. Full list of identified lipid species in four developmental stages/sexes [i.e. egg, third-stage (L3) larvae; female (AF) and male (AM) adults] of *Nippostrongylus* *brasiliensis*.

Additional file 2: Figure S1. Quantitative differences in MG, DG, PA, PG, PI, PS, CL, LPC, LPE, LPG, LPI, LPS and SM lipids among four developmental stages/sexes of *Nippostrongylus* *brasiliensis*.

Additional file 3: Table S2. Results of statistical analyses performed using a one-way ANOVA post hoc test.

## Data Availability

No datasets were generated or analysed during the current study.

## Abbreviations

GL: Glycerolipid
GP: Glycerophospholipid
SL: Sphingolipid
MG: Monoradylglycerols
DG: Diradylglycerols
TG: Triradylglycerols
PC: Glycerophosphocholines
PE: Glycerophosphoethanolamines
PG: Glycerophosphoglycerols
PI: Glycerophosphoinositols
PS: Glycerophosphoserines
CL: Cardiolipins, For LPC, LPE, LPG, LPI and LPS, prefix 'L' was added for each lysoglycerophospholipid class
SM: Sphingomyelins
Cer: Ceramide
HexCer: Hexosylceramide
CE: Cholesteryl ester
FA: Fatty acyl
RSD: Relative standard deviation
