# Supplementary material for: The developmental lipidome of Nippostrongylus brasiliensis
Source: Parasit Vectors. 2025 Jan 25;18:27. doi: 10.1186/s13071-024-06654-2 (PMC11762861; doi:10.1186/s13071-024-06654-2)
Supplement: Supplementary file 2 — Additional file 2: Figure S1. Quantitative differences in MG, DG, PA, PG, PI, PS, CL, LPC, LPE, LPG, LPI, LPS and SM lipids among four developmental stages/sexes of Nippostrongylus brasiliensis. [file 13071_2024_6654_MOESM2_ESM.docx]

SUPPLEMENTARY INFORMATION:

**Figure S1.** Quantitative changes of MG, DG, PA, PG, PI, PS, CL, LPA, LPC, LPE, LPG, LPI, LPS and SM lipids in different developmental stages/sexes of *Nippostrongylus* *brasiliensis*. Four developmental stages/sexes include egg, third-stage (L3) larvae; female (AF) and male adults (AM). Statistical analysis was performed by ANOVA (only significant results with **** *P* < 0.0001; details were provided in Additional file 2: Table S3). Error bars indicate standard deviation (four replicates).
